# Native Nephrectomy before and after Renal Transplantation in Patients with Autosomal Dominant Polycystic Kidney Disease (ADPKD)

**DOI:** 10.3390/jcm8101622

**Published:** 2019-10-04

**Authors:** Andreas Maxeiner, Anna Bichmann, Natalie Oberländer, Nasrin El-Bandar, Nesrin Sugünes, Bernhard Ralla, Nadine Biernath, Lutz Liefeldt, Klemens Budde, Markus Giessing, Thorsten Schlomm, Frank Friedersdorff

**Affiliations:** 1 Department of Urology, Charité–Universitätsmedizin Berlin, Corporate Member of Freie Universität Berlin, Humboldt-Universität zu Berlin, and Berlin Institute of Health, Charitéplatz 1, 10117 Berlin, Germany; Andreas.maxeiner@charite.de (A.M.); natalie.oberlaender@hotmail.de (N.O.); nasrin.el-bandar@charite.de (N.E.-B.); nesrin.suguenes@charite.de (N.S.); bernhard.ralla@charite.de (B.R.); nadine.biernath@charite.de (N.B.); thorsten.schlomm@charite.de (T.S.); 2 Department of Anesthesiology and Operative Intensive Care Medicine, Campus Charité Mitte, Charité–Universitätsmedizin Berlin, Corporate Member of Freie Universität Berlin, Humboldt-Universität zu Berlin, and Berlin Institute of Health, Charitéplatz 1, 10117 Berlin, Germany; anna.bichmann@charite.de; 3 Department of Nephrology, Charité–Universitätsmedizin Berlin, Corporate Member of Freie Universität Berlin, Humboldt-Universität zu Berlin, and Berlin Institute of Health, Charitéplatz 1, 10117 Berlin, Germany; lutz.liefeldt@charite.de (L.L.); klemens.budde@charite.de (K.B.); 4 Department of Urology, Heinrich-Heine-University, 40225 Düsseldorf, Germany; markus.giessing@med.uni-duesseldorf.de

**Keywords:** ADPKD, native nephrectomy, kidney transplantation, patient outcome, perioperative complications

## Abstract

The aim of this study was 1) to evaluate and compare pre-, peri-, and post-operative data of Autosomal Dominant Polycystic Kidney Disease (ADPKD) patients undergoing native nephrectomy (NN) either before or after renal transplantation and 2) to identify advantages of optimal surgical timing, postoperative outcomes, and economical aspects in a tertiary transplant centre. This retrospective analysis included 121 patients divided into two groups—group 1: patients who underwent NN prior to receiving a kidney transplant (*n* = 89) and group 2: patients who underwent NN post-transplant (*n* = 32). Data analysis was performed according to demographic patient details, surgical indication, laboratory parameters, perioperative complications, underlying pathology, and associated mortality. There was no significant difference in patient demographics between the groups, however right-sided nephrectomy was performed predominantly within group 1. The main indication in both groups undergoing a nephrectomy was pain. Patients among group 2 had no postoperative kidney failure and a significantly shorter hospital stay. Higher rates of more severe complications were observed in group 1, even though this was not statistically significant. Even though the differences between both groups were substantial, the time of NN prior or post-transplant does not seem to affect short-term and long-term transplantation outcomes. Retroperitoneal NN remains a low risk treatment option in patients with symptomatic ADPKD and can be performed either pre- or post-kidney transplantation depending on patients’ symptom severity.

## 1. Introduction

Autosomal dominant polycystic kidney disease (ADPKD) is the fourth most frequent cause of end-stage renal disease (ESRD) in Europe, accounting for around 10–15% of patients on dialysis and 9% to 10% of renal transplantation [1,2]. ADPKD patients develop progressive expansion of multiple bilateral cysts in the renal parenchyma, causing a deterioration of their glomerular filtration rate (GFR) [3]. Patients with ADPKD often develop recurrent urinary tract infections, nephrolithiasis, and back or abdominal pain. Approximately one-fifth of ADPKD patients will require unilateral or bilateral nephrectomy at some point in their life [4,5,6]. Due to a heterogeneous clinical presentation ranging from asymptomatic to very severe, treatment options are highly variable. In comparison to other forms of renal replacement therapy, kidney transplantation seems to be the option of choice in most ESRD patients, with improved survival and lower morbidity [7]. However, the optimal time for nephrectomy in ADPKD patients awaiting renal transplantation remains a matter of debate. Furthermore, the severity of clinical symptoms may also influence patients’ wishes to undergo nephrectomy. Some previously published research does not recommend pre-transplant nephrectomy due to associated increased morbidity and mortality [8,9]. Others suggest a “sandwich technique”, whereby the most severely affected native kidney is removed before, and the remaining polycystic kidney is removed after transplantation [10,11]. Concomitant nephrectomy and transplantation is another method that is described within the literature [6,12], which is predominantly used for ADPKD patients who are scheduled for living donor kidney transplants. The aim of this study was (1) to evaluate and compare pre-, peri-, and post-operative data of ADPKD patients undergoing native nephrectomy either before or after renal transplantation and (2) to identify advantages of optimal surgical timing, postoperative outcomes, and economical aspects in a tertiary transplant center.

## 2. Methods

### 2.1. Patient and Study Design

The retrospective analysis included 141 patients with ADPKD who underwent unilateral surgical nephrectomy between January 2005 and December 2018. Twenty patients were excluded due to incomplete data. Three patients underwent bilateral nephrectomy sequentially and were also excluded. Group 1 included nephrectomy patients who were on dialysis prior to kidney transplantation (*n* = 89) and group 2 represents patients who had post-transplant nephrectomy (*n* = 32). Data analysis was performed according to demographic patient details, surgical indication, laboratory parameters, perioperative complications, underlying pathology, and associated mortality. Patients in group 2 received a standard triple maintenance immunosuppression that consisted of tacrolimus or cyclosporin A in combination with mycophenolate mofetil and prednisolone.

### 2.2. Surgical Procedure

**Figure 1:**
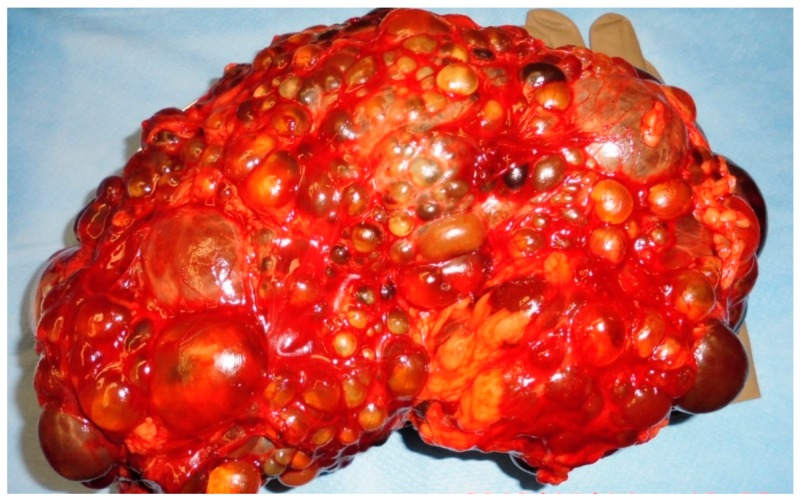
Polycystic kidney preparation after retroperitoneal nephrectomy.

The operation procedure was performed by a unilateral flank incision of 20–25 cm with perioperative antibiotic treatment. A strictly extra-peritoneal surgical preparation was performed. If an intraoperative peritoneal laceration occurred, an immediate surgical reparation was done. The vessel hilum was sealed by using three Hem-o-lok clips. Surgical drains were placed at the time of transplant and were present postoperatively. Figure 1 shows a removed polycystic kidney preparation after retroperitoneal nephrectomy.

### 2.3. Statistical Analysis

Statistical analyses were performed using SPSS (SPSS Inc., version 25, Armonk, NY, USA). Both univariate and multivariate analyses were applied to identify risk factors for complications following cystic kidney removal, both before and after kidney transplantation. Baseline characteristics were compared using the Chi-squared test and Fisher’s exact test for categorical variables. Continuous variables were tested with the Student’s t-test or Mann–Whitney U-test (if the assumption of Gaussian distribution was not fulfilled). Results were reported as means and standard deviations (SD) for continuous variables; categorical variables were reported as numbers and percentages. For all the statistical measures, a *p*-value <0.05 was considered significant. Odds ratio (OR) was calculated and statistical determinations were within the 95% confidence interval. 

## 3. Results

### 3.1. Demographic Data

Out of the 121 included patients with ADPKD, 89 patients underwent nephrectomy prior to kidney transplantation (group 1) and 32 patients underwent nephrectomy post-transplant (group 2). Patient’s demographic data is displayed in Table 1 below.

There was no significant difference in the patient demographics between both groups, although right-sided nephrectomy was predominantly performed within group 1 (*p* = 0.02). The main comorbidities in both groups were cardiovascular diseases (group 1: 83.1% verus group 2: 81.3%; *p* = 0.808), which were represented most commonly by coronary artery disease, hypertension, and peripheral vascular disease.

### 3.2. Indications

Table 2 shows the individual indications for a nephrectomy.

### 3.3. Patient Outcome Analysis

Comparing pre-operative serum creatinine levels in patients within group 2, a mild increase from an average pre-operative level of 1.47 mg/dL to 1.61 mg/dL postoperatively occurred. No patients had peri-operative kidney failure. In all 32 cases undergoing post-transplantation nephrectomy, the initially elevated creatinine levels postoperatively appeared stable (3 months: 1.62 mg/dL, 6 months: 1.64 mg/dL, 1 year: 1.69 mg/dL, and 3 years: 1.64 mg/dL). The difference between the pre- and post-operative haemoglobin levels was insignificant (group 1: 2.2 g/dL versus group 2: 2.5 g/d, *p* = 0.468). The difference in surgical time between both groups was insignificant (group 1: 175 min versus group 2: 170.5 min, *p* = 0.541), although a significant difference in the duration of hospital admission was observed (group 1: 7 days versus group 2: 6 days; *p* = 0.001). The pathological assessment of polycystic nephrectomy samples showed a 3% risk for renal cell carcinoma in both groups (group 1: 3.4% versus group 2: 3.1%; *p* = 1.0). No statistical difference was reported in the rates of acute inflammation in the pathological report (group 1: 15.6% versus group 2: 5.6%; *p* = 0.127). Furthermore, there was no significant difference between the chronic renal inflammation rates (group 1: 61.8% versus group 2: 71.9%; *p* = 0.307), which were defined as low-grade chronic systemic inflammation characterized by persistent, low to moderate levels of one or more circulating inflammation markers, such as white blood cells count, C-reactive protein, and procalcitonin. However, a significant difference was observed in the median weight of the removed kidney (group 1: 2600 g compared to 1683 g in group 2 (*p* = 0.004)). Concerning postoperative complication rates, group 1 had a higher prevalence of 43.8% compared to 37.5% within group 2, even though it was not statistically significant (*p* = 0.936). The complications within group 1 were classified as Clavien-Dindo 1 in 7.9% and as Clavien-Dindo 2 in 22.5%. Those categorized as Clavien-Dindo 3 (7.9%) included two patients suffering from a pneumothorax and one patient appeared with a pancreatic injury. Severe complications (Clavien-Dindo 4: 5.5%) included two patients of whom one required laparotomy on the second postoperative day due to a retroperitoneal abscess and one suffered a pulmonary embolism with subsequent cardiac arrest with a return of spontaneous circulation upon resuscitation. A total of three patients died within group 1 (3.4%), of which two suffered severe sepsis and one a hypoglycaemic shock. Complication rates within group 2 were mostly minor (Clavien-Dindo 1: 9.4%, Clavien-Dindo 2: 25%). Only one patient was classified as Clavien-Dindo 3. No patients in group 2 were categorized as Clavien-Dindo 4 or deceased. Among all outcome parameters, the multivariate analysis identified the following parameters as significant risk factors for a prolonged hospital stay: age (*p* <0.001) as well as undergoing native nephrectomy prior to transplantation (represented by group 1) (*p* = 0.013). Factors such as male sex, body mass index (BMI), organ weight, duration of operation, and time on dialyses were not significant risk factors for a prolonged hospital stay. 

## 4. Discussion

ADPKD is the most common inherited disease with over 12 million patients with associated terminal renal failure, representing the fourth leading cause for dialysis worldwide. Patients suffering from ADPKD develop progressive expansion of multiple bilateral cysts in the renal parenchyma, causing a deterioration of GFR [3]. Patients with ADPKD often develop recurrent urinary tract infections, nephrolithiasis, and back or abdominal pain. Approximately one-fifth of ADPKD patients will require unilateral or bilateral nephrectomy at some point in their life [4,5,6]. It is still unclear if patients undergoing nephrectomy post-transplant have higher complication rates. This research was not able to display a significant difference between both groups, even though the prevalence of complications was higher within group 1 (43.8% versus 37.5%).

The indications for nephrectomy were often multiple for each patient and the major indications are listed in Table 2. Overall, the main nephrectomy indication was pain in over 50% of the patients. Further indications for nephrectomy need to be critically evaluated and can be based on intra-abdominal space issues, uncontrolled hypertension, and cystic bleeding [6,7,13]. Due to the often-present additional liver cysts in 80% of patients with ADPKD [14], a right-sided intra-abdominal space problem occurs, resulting in less affected contralateral kidneys. The statistically significant difference in organ weight, as stated previously (2600 g versus 1683 g, *p* = 0.004), seems to underline this hypothesis. In addition, kidney volume seems to be an early marker of severity of the disease and is shown to be a determinant of a reduction in kidney functions [3].

According to the literature, no difference in average age at the time of nephrectomy (*p* = 0.927) was observed [7,15] and, further, a higher rate of male patients was also reported in our cohort despite being an autosomal dominant disease [6,13,15]. Lifestyle and preventive factors also need to be addressed in ADPKD. Patients can prevent disease progression by controlling hypertension through a low salt diet [16,17]. These statements are of limited value for external validity as these publications involved low case numbers of patients with ADKPD who were reviewed at the time of nephrectomy. Nevertheless, the assumption can be made that disease progression can be delayed by a healthy lifestyle. Studies have shown that females have higher health awareness in their daily living [18], which could also explain the male dominant cohort. However, cyst expansion can cause ischemia within the kidney and, consequently, the activation of Renin-Angiotensin-Aldosterone-System (RAAS), leading to the development and/or maintenance of hypertension [19]. Hence, patients might benefit from native nephrectomy if hypertension is predominantly present. On the other hand, preserving patient’s urine excretion might also preserve quality of life concerning daily fluid intake.

Research published focusing on patients who are positive for polycystic kidney disease 1 (PKD1) mutations prior to the age of 35 years show a worse and faster disease progression in the male sub-cohort and/or hypertension [20]. However, the average BMI within our cohort was around a normal range of 25 kg/m^2^. A known disadvantage using BMI as a reference is the inconsideration of water and muscle mass, which is greatly variable in these patients. Hence, observing that the BMI does not increase in pre-transplant patients despite significant edemas has not yet been discussed in published research.

In all cases, only unilateral nephrectomy was carried out. This changed to bilateral nephrectomies prior to renal transplants in the 1970s and reduced infection-based complications [21]. Nevertheless, higher postoperative complications were observed, including worsening anemia and loss of diuresis [22]. Within the past few decades, bilateral nephrectomy case numbers decreased due to advanced medication and stricter surgical indication. Fuller et al. reviewed a small cohort of 32 patients who underwent simultaneous and sequential bilateral nephrectomy [6]. Out of the studied 25 patients, 6 had simultaneous bilateral native nephrectomy with higher rates of blood transfusions, increasing antibody production, and worsening post-transplant outcomes [6]. Hence, the authors concluded not to promote bilateral simultaneous intervention. Further, 3% of patients in both groups were found to have a histological diagnosis of coincidental cancer, which is in concurrence with literature published to date [6,7,13]. Histological analysis within the post-transplant group showed higher rates of inflammation, which is potentially due to the effects of immunosuppressive medication. These findings have not been published elsewhere. We suggest taking samples from potentially inflamed cysts intraoperatively. An extended antibiotic cover with lipophilic properties can then be discussed. 

However, our study found no significant differences between patients undergoing native nephrectomy prior or post-transplant. Hence, the role of transplantation and subsequent immunosuppressive therapy seems to be irrelevant as group 2 had less severe and a smaller number of post-operative complications. The deceased patients within group 1 were patients without transplants. Chebib et al. had fewer complications in the cohort undergoing a nephrectomy post-transplant [15]. Similar observations were published by Kirkman et al. [7]. Thus, the presumed increased risk through immunosuppressive therapy concerning wound healing and increased infection rates post-operatively cannot be supported. The significantly shorter hospital stay of the post-transplant patients in our cohort also represents a fact that can be witnessed within the literature [6]. 

Despite our findings, we acknowledge limitations of the present study and potential sources of bias that need to be addressed. The retrospective analysis as well as the limited number of patients of group 2 might confound our results. Furthermore, the exclusion of 23 patients due to missing data may have also decreased the potential study cohort. In addition, analysis of short-term and long-term transplantation outcomes (graft loss, delayed graft function, acute rejection, bacterial and cytomegalovirus (CMV] infection, and post-transplant diabetes mellitus) between both groups was not included.

## 5. Conclusions

In conclusion, our study demonstrates that open retroperitoneal nephrectomies represent a low risk management option in patients with symptomatic ADPKD and post-transplant nephrectomy seems to not be associated with higher complication rates. Hence, timing and indication of native nephrectomy should be primarily based on symptom severity rather than on the date of transplantation.

## Figures and Tables

**Table 1:** Demographic data.

Parameter	Group 1 (*n* = 89)	Group 2 (*n* = 32)	*p*-Value
Age average in years	53.92	53.75	0.927
Male sex (%)	69.70	68.80	0.923
Right sided nephrectomy (%)	58.40	34.40	0.02 *
Left sided nephrectomy (%)	41.6	65.6	0.02 *
BMI (kg/m^2^average)	25.93	25.31	0.445
Median duration of dialysis (months)	33.00	22.00	0.100
Median weight of the removed kidney	2600 g	1683 g	0.004

Group 1: pre-transplant, Group 2: post-transplant. *, statistically significant; BMI, body mass index.

**Table 2:** Indications for a nephrectomy.

Indications	Group 1 (*n* = 89)	Group 2 (*n* = 32)	*p*-Value
Renal pain (%)	50.6	59.4	0.392
Infection (%)	31.5	28.1	0.725
Urolithiasis (%)	11.2	6.3	0.514
Haematuria (%)	4.5	6.3	0.654
Gastrointestinal complaints (%)	2.2	0	1.000

Group 1: pre-transplant, group 2: post-transplant.
